# A Layer-Dependent Analytical Model for Printability Assessment of Additive Manufacturing Copper/Steel Multi-Material Components by Directed Energy Deposition

**DOI:** 10.3390/mi12111394

**Published:** 2021-11-13

**Authors:** Wenqi Zhang, Baopeng Zhang, Haifeng Xiao, Huanqing Yang, Yun Wang, Haihong Zhu

**Affiliations:** 1 Wuhan National Laboratory for Optoelectronics, Huazhong University of Science and Technology, Wuhan 430074, China; vinkyz@hust.edu.cn (W.Z.); zhangbp@hust.edu.cn (B.Z.); xiaohaif@hust.edu.cn (H.X.); 2 XI’AN Space Engine Company Limited, Xi’an 710100, China; 13991882146@163.com (H.Y.); wyun7103@163.com (Y.W.)

**Keywords:** directed energy deposition, additive manufacturing, bimetal, analytical model, printability maps

## Abstract

Copper/steel bimetal, one of the most popular and typical multi-material components (MMC), processes excellent comprehensive properties with the high strength of steel and the high thermal conductivity of copper alloy. Additive manufacturing (AM) technology is characterized by layer-wise fabrication, and thus is especially suitable for fabricating MMC. However, considering both the great difference in thermophysical properties between copper and steel and the layer-based fabrication character of the AM process, the optimal processing parameters will vary throughout the deposition process. In this paper, we propose an analytical calculation model to predict the layer-dependent processing parameters when fabricating the 07Cr15Ni5 steel on the CuCr substrate at the fixed layer thickness (0.3 mm) and hatching space (0.3 mm). Specifically, the changes in effective thermal conductivity and specific heat capacity with the layer number, as well as the absorption rate and catchment efficiency with the processing parameters are considered. The parameter maps predicted by the model have good agreement with the experimental results. The proposed analytical model provides new guidance to determine the processing windows for novel multi-material components, especially for the multi-materials whose physical properties are significantly different.

## 1. Introduction

Multi-material components (MMC), such as gradient materials, dissimilar joints, and sandwich structure materials, are characterized by spatial composition variation in one or more directions [1]. Due to their unique properties with progressive change in performance and function, MMC has gained notable attention and has been widely used in many fields such as electrical and aerospace over the past few decades [2]. Copper/steel bimetal, one of the most popular and typical MMC, processes excellent comprehensive properties with the high strength of steel and the high thermal conductivity of copper alloy. Because of its excellent properties, copper/steel has found its applications in the power generation, transmission, and die-casting industries [3]. Despite its attractive function and thermophysical properties, fabricating copper/steel MMC is still challenging due to its heterogeneous materials and thermophysical properties [4].

Additive manufacturing (AM) has been identified as an innovative manufacturing method that enables the build-up of components with complex geometries directly from 3D models. AM technology is characterized by the layer-wise fabrication of a part through selectively adding and melting material. It provides many advantages, including high manufacturing freedom, excellent part performance, and high production efficiency [5]. Due to the layer-wise process approach, AM is especially suitable for fabricating MMC [6,7].

**Figure 1:**
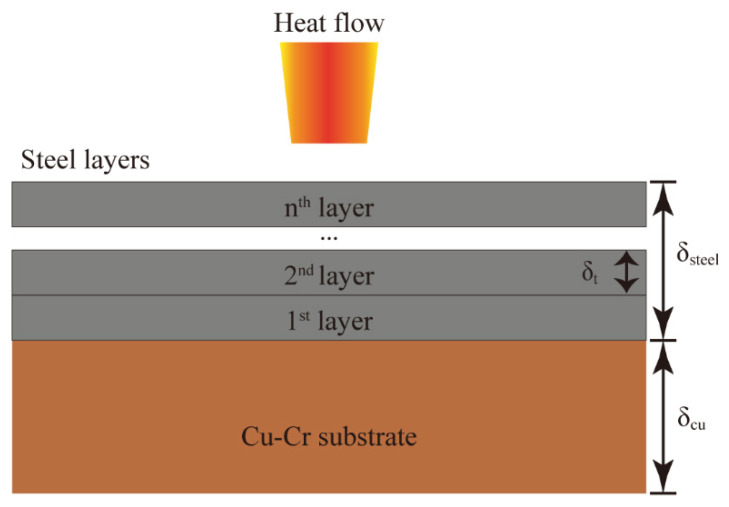
The schematic of fabricating the steel on the CuCr substrate layer by layer, where δ_cu_, δ_steel_, and δ_t_ represent the thickness of the CuCr substrate, the total thickness of steel coatings and the thickness of single layer steel coating, respectively.

With the growing requirement in industrial applications, many researchers are committed to using AM methods to fabricate MMC. The two most popular AM technologies, directed energy deposition (DED) [8,9] and powder bed fusion (PBF) [10,11], have been widely investigated to fabricate the single material including copper alloys [12,13,14], iron alloys [15,16,17,18], titanium alloys [19,20,21], aluminum alloys [22,23,24,25], and nickel alloys [26,27]. However, the AM process for heterogeneous materials is very different from that for homogeneous materials. In general, processing parameters (for example, laser power and scanning velocity) and material properties (for example, thermal conductivity, specific heat capacity, and density) influence the thermal profile and the printability of the AM process. Because effective thermal conductivity varies between layers, the processing parameters may also need to change as the deposition layer numbers increase. This phenomenon is particularly prominent for copper/steel dissimilar materials, because the thermophysical properties, such as thermal conductivity, of copper and steel differ greatly. The schematic of fabricating steel on the CuCr substrate is illustrated in Figure 1.

When investigating the existing literature on AM of fabrication copper/steel or steel/copper bimetal, processing parameters optimization for every layer is absent. At present, there are mainly two methods to fabricating dissimilar materials. One common method is to use fixed parameters for steel and copper, respectively. Bai et al. [28] manufactured 316L/C52400/316L sandwich structure materials by SLM, and used the parameters for C52400 copper alloy and 316L, separately. Liu et al. [29] and Chen et al. [30] used different processing parameters for the individual alloys of steel and copper. The other common method is to utilize variable parameters, i.e., one set of parameters for the interfacial layers of copper/steel and another set of parameters for steel or copper, respectively. Chen et al. [31] optimize the interfacial layers by orthogonal experiment, and the set of parameters are fixed for steel and copper, respectively. Tan et al. [32] successfully processed steel on the copper alloy substrate by SLM, using one set of parameters for the first ten layers by remelting twice and using the optimized parameters of steel for the rest of the fabricating. In our previous work [3], the steel was built on the CuCr alloy substrate by DED. We optimize the parameters from one to four layers and the optimized parameters for steel are used for the rest of the layers. Based on the literature discussed above, there is currently no known work describing process-layer number relationships for fabricating copper/steel bimetal in the contest of AM.

Considering both the great difference in thermophysical properties between copper and steel and the layer-based fabrication character of the AM process, the optimal processing parameters will vary throughout the deposition process. Since there are numerous process variables within AM, the optimization of processing parameters for every layer is a huge amount of work [33]. Therefore, establishing a framework to implement model-based approaches to building dissimilar material parts is essential.

In this paper, we propose an analytical calculation model to predict the layer-dependent processing parameters during the fabrication of copper/steel bimetal. Specifically, the changes in effective thermal conductivity and specific heat capacity with the layer number as well as the absorption rate and catchment efficiency with the processing parameters are considered. The analytical model is established to predict molten pool temperature and thus to provide a methodology to estimate the process maps for multi-layer copper/steel bimetal dependent on the layer number. These results are compared with experimentally observed molten pool width and parameter maps for copper/steel specimens and found to have good agreement.

## 2. Theoretical Modeling

**Figure 2:**
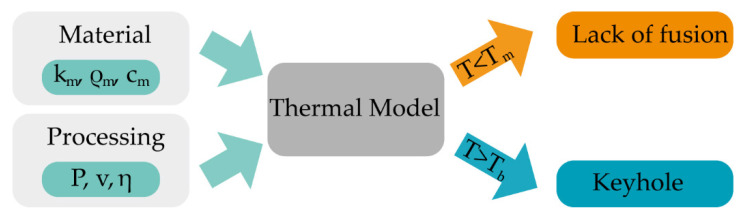
The framework for estimating the layer-dependent printability of the copper/steel bimetal. Processing parameters (laser power (P), scanning velocity (v), and powder catchment efficiency (η)) and layer-dependent effective material properties [35] (effective thermal conductivity (k_m_), effective specific heat (c_m_), and effective density (ρ_m_)) are provided to the thermal model.

Figure 2 shows the framework for estimating the layer-dependent printability of the copper/steel bimetal. The general workflow starts with the calculations of layer-dependent thermophysical properties (the effective thermophysical properties [34]) and the processing parameters-dependent catchment efficiency [35]. Then the temperature fields and molten pool dimensions with different laser power and scanning velocity covering the processing space are obtained. The peak temperature (T_max_) and dimensions of the molten pool are subsequently used to evaluate the parameter maps and verify the model. In this study, the stainless steel is fabricated on the CuCr substrate, hence regions of process space with T_m_steel_ < T_max_ < T_b_cu_ are recognized to be the appropriate combination of processing parameters for the first layer, and T_m_steel_ < T_max_ < T_b_steel_ for the second layer and above, where T_m_steel_, T_b_steel_, T_m_cu_, and T_m_cu_ represent the melting point and boiling point of steel and Cu, respectively. The peak temperature criterion has also been applied in the selective laser melting process [36]. Details of each step in this workflow are presented in the subsequent sections.

### 2.1. Thermal Model

In this framework, the temperature profile is required to calculate the temporally evolving molten pool dimensions and the temperature at the molten pool boundary (i.e., the solid-liquid interface) and the center (i.e., T_max_). The assumption that the solidification process occurs at a constant temperature is reasonable because of the high thermal gradient and cooling rate for DED.

The Cline–Anthony model [37] is used to calculate the temperature field of the molten pool during the DED process. The Cline–Anthony model was established to analyze the thermal distribution and geometry of melting track from a moving Gaussian source on a semi-infinite substrate. Since the laser beam in this paper is small compared to the substrate, a semi-infinite geometry is a rationally good approximation. Therefore, the Cline–Anthony model is applicable in this paper. The Cline–Anthony model is beneficial to rapidly handle many calculations throughout the entire processing maps and has been widely used in the AM process [38,39]. The temperature field of the molten pool relating to the processing parameters and the thermophysical properties during DED can be calculated by the Cline–Anthony model as Equation (1):

(1) $$T\left(x,y,z\right)=T_{0}+\frac{P_{e}}{{\left(2\pi^{3}\right)}^{1/2}k_{m}r}\int_{0}^{\infty }\frac{1}{1+s^{2}}\exp\left\{-\frac{1}{2\left(1+s^{2}\right)}\left[{\left(\frac{x}{r}+s^{2}ar\right)}^{2}+{\left(\frac{y}{r}\right)}^{2}\right]-\frac{1}{2s^{2}}{\left(\frac{z}{r}\right)}^{2}\right\}ds$$

where *T*_0_ is to the initial temperature of the substrate, *P_e_* is the total laser power absorbed by the powder and the substrate, r is the radius of the laser spot, *k_m_* is the effective thermal conductivity of the substrate, *a* is defined as *a* = ρ_m_c_m_v/(2 k_m_), and s is defined as *s*^2^ = 2 *k_m_*t/(ρ_m_c_m_r) (ρ_m_, c_m_, v, and t refer to the density, specific heat capacity of the MMC, laser scanning speed, and laser scanning time, respectively).

**Figure 3:**
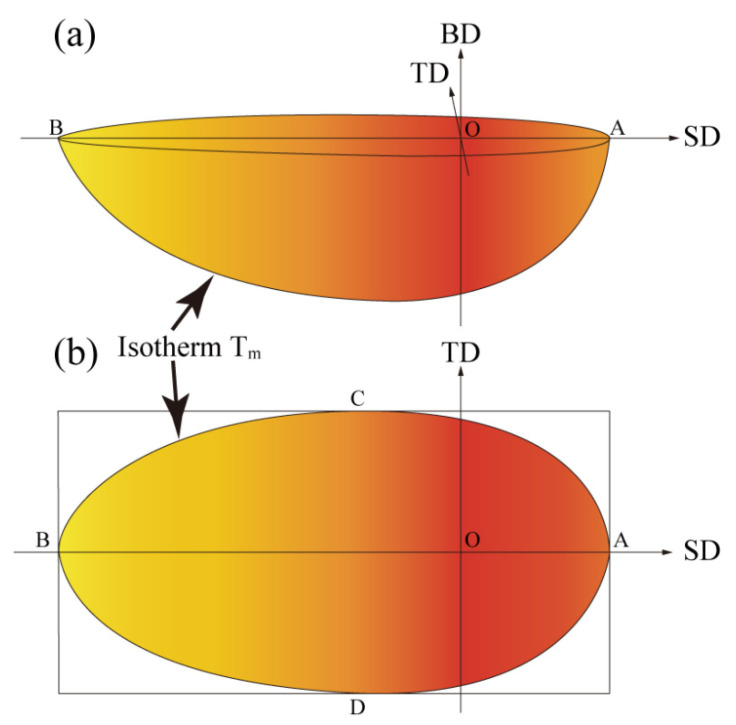
Schematic of the molten pool (SD, TD, BD refer to scanning, transverse, and build directions, respectively): (**a**) 3D diagram of the molten pool; (**b**) SD-TD cross-section of the molten pool.

The molten pool dimension is figured by the zone enclosed by the isosurface of the solidification temperature or melting point (T_m_) as illustrated in Figure 3. Figure 3a shows the 3D diagram, where SD, TD, and BD refer to scanning, transverse, and build directions, respectively. Figure 3b shows the SD-TD cross-section. Then the width (W) and length (L) of the molten pool can be obtained, where W equals the length of CD, and L equals the length of AB.

### 2.2. The Laser–Materials Interaction during the DED Process

**Figure 4:**
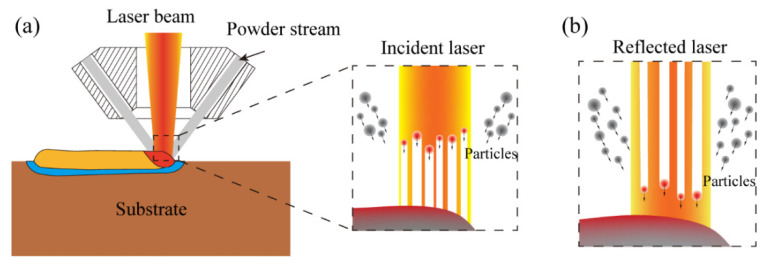
Schematic of the laser–material interaction: (**a**) the powder interacts with the incident laser; (**b**) the powder interacts with the reflected laser.

The typical shadow model is used for the laser–materials interaction during the DED process [38,40]. Figure 4 describes the laser–materials interaction during the DED process of fabricating steel on the copper substrate. The laser beam, metal powder, and protective gas are output from the nozzle coaxially. When traversing the powder stream, part of the laser beam interacts with the powder. Therefore, a portion of laser energy is attenuated (absorbed or scattered) by the powder, and the rest reaches the substrate surface [38]. However, due to the high reflectivity of the copper alloy substrate, a large portion of energy reaching the substrate is reflected and can be absorbed by the powder again. Finally, the energy carried by the heated powder particles partially falls into the molten pool for further melting. As the laser leaves, the molten pool cools and solidifies rapidly on the substrate surface to form the deposition track. The following assumptions are made for the usage of the proposed model during the DED process [40]:

(1) The laser energy attenuation is proportional to the projected area of the powder particles in the laser beam. Since the powder concentration is much smaller compared with the gas flow volume, it is reasonable to neglect the shadow between particles.
(2) The powder particles are considered homogeneous and spherical. The average diameter is used to represent the particle size. The argon gas atomized powder is used in this work, and the morphology of the powders is almost spherical from the scanning electron microscope (SEM) observation [3].
(3) The thermophysical properties of materials are regarded as constant and invariable with the temperature.
(4) The laser beam reaching the substrate is perfectly reflected upwards in the same shape as the initial beam.

Based on the above assumptions, the attenuation rate of the laser energy by the powder particles can be obtained, which equals the ratio between the projected area of the powder particles and the laser beam. First, the laser beam travels through the gas–powder stream and is attenuated (absorbed or scattered) before reaching the substrate. The attenuate ratio β_att_ can be expressed as Equation (2), which is the proportion of the projected area of the powder particles to the laser beam area [38].

(2) $$\beta_{att}=\frac{S_{p}}{S_{l}}=\frac{3m_{p}}{2\pi \rho_{p}r_{p}rv_{p}cos\theta }$$

where *S_p_* is the projected area of the powder particles, *S_l_* is the projected area of the laser beam, *m_p_* (kg/s) is the powder feeding rate, *ρ_p_* (kg/m^3^) is the density of the powder, *r_p_* (m) is the average diameter of the powder particles, *r* (m) is the laser beam radius, *v_p_* (m/s) is the velocity of the powder-gas stream, and *θ* is the angle between the gas–powder and the horizontal. The attenuated laser power (*P_att_*) by the powder can be obtained as Equation (3):

(3) $$P_{att}=\beta_{att}*P$$

where *P* is the laser power. A portion of the attenuated laser energy is absorbed by the powder stream and delivered to the substrate when the particles enter the molten pool. The absorbed laser power by the powder stream delivered to the substrate can be expressed as Equation (4).

(4) $$P_{a1}={\eta *\beta }_{p}*P_{att}$$

where β*_p_* is the laser absorption of the powder and η the powder catchment efficiency. Second, the laser beam passes through the gas–powder stream and reaches the substrate. Then the substrate can absorb part of the laser energy (*P_s_*) directly based on the laser absorptivity, as is given by Equation (5):

(5) $$P_{s}=\beta_{s}(P-P_{att})$$

where β*_s_* is the laser absorption of the substrate. Finally, the laser beam reflected by the substrate is important for the high laser reflectivity of CuCr alloy substrate. Therefore, part of the reflected laser energy (*P_r_*) is attenuated and absorbed by the powder stream again. The powder stream transfers the absorbed energy back to the substrate (*P_a_*_2_), as is given by Equations (6) and (7):

(6) $$P_{a2}=\eta {\;*\;\beta }_{att}{\;*\;\beta }_{p}\;*\;P_{r}$$

(7) $$P_{r}=(1-\beta_{s})(P-P_{att})$$

Thus, the effective laser power (*P_e_*), namely the total amount of laser power delivered into the molten pool by the powder stream and absorbed by the substrate, can be obtained by Equation (8):

(8) $$P_{e}=P_{a1}+P_{s}+P_{a2}\;$$

### 2.3. The Catchment Efficiency

From Equations (4), (6), and (8), the powder catchment efficiency (η) is essential for the calculation of the temperature field. According to reference [35], the defined parameter *Q*, which is related to the processing parameters and material properties, can be used to evaluate the catchment efficiency as expressed by Equation (9):

(9) $$Q=\frac{{\left(P/v\right)}^{2/3}}{{\left(c\Delta T+H\right)}^{2/3}}$$

where Δ*T*, *H*, and *c* refer to the difference between the solidus temperature and ambient temperature, the latent heat of fusion, and the specific heat capacity of the alloy, respectively. For the convenience of comparing different processing parameters, the normalized value Q*, which is expressed as *Q* divided by *Q*_max_ (the maximum value for all the data used), is used to calculate the value of η as Equation (10) [35]:

(10) $$\eta =-1.5{\left(Q^{*}\right)}^{2}+2.8\left(Q^{*}\right)-0.3$$

### 2.4. Evolution of the Substrate Laser Absorption

**Figure 5:**
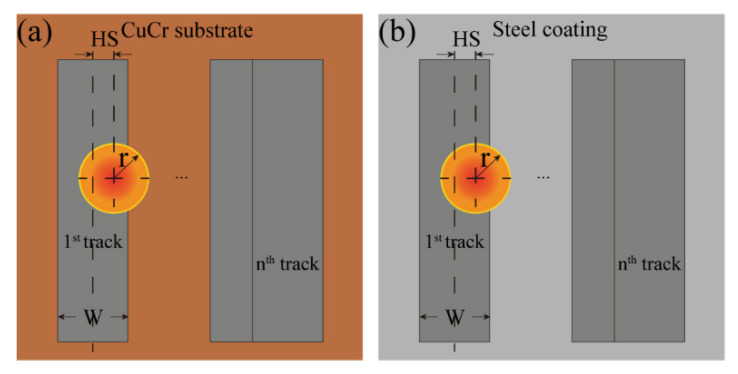
Evolution of the substrate laser absorption: (**a**) the first layer except for the first track; (**b**) the second layer and above.

The laser absorption of the substrate varies with the track and layer numbers for the heterogeneous materials. For the first track of the first layer, the laser interacts with the copper alloy substrate, and the absorption of the laser by the substrate (β_s_) equals the copper substrate β_s_ = β_cu_, where β_cu_ represents the laser absorption of the CuCr alloy. For the second track and above the first layer, part of the laser interacts with the previous steel track, and the rest with the substrate directly as shown in Figure 5. It is considered that the absorption of the substrate is the linear sum of the laser absorption rate of copper and steel and expressed as Equation (11):

(11) $$\beta_{s}=\left(1-l\right)*\beta_{cu}+l*\beta_{steel}\;$$

where *β_steel_* represents the laser absorption of the steel, *l* is related with the laser radius (r), the width of the previous track (W), and the hatching space (HS) and can be expressed as Equation (12):

(12) $$l=\frac{r+\left(\frac{W}{2}-HS\right)}{2r}\;$$

For the second layer and above, the laser interacts with the steel coating directly, and the absorption of the laser by the substrate can be expressed as β_s_ = β_steel_ as shown in Figure 5b.

### 2.5. Evolution of the Effective Thermophysical Properties

The thickness of copper and steel, which is a function of the deposition layer number, determines the thermophysical properties of the copper/steel bimetal as a single component. Equation (13) gives the thermal resistance of a single material, where R, δ, k, and A indicate the thermal resistance, the thickness of the material, the thermal conductivity, and the area of the cross-section that is perpendicular to the heat flow direction, respectively. The total thermal resistance (*R_m_*) of the MMC, as seen in Figure 1, can be represented in Equation (14). The subscript m, steel, and cu denote the copper/steel MMC, the stainless steel, and CuCr alloy, respectively. If the copper/steel bimetal is treated as a single component, the theoretical effective thermal conductivity [34] (*k_m_*), density (*ρ_m_*), and specific heat capacity (*c_m_*) of the MMC are obtained in Equations (15)–(17) [41,42]. Where $V_{steel}^{\prime }=\frac{V_{steel}}{V_{steel}+V_{cu}}$, $V_{cu}^{\prime }=\frac{V_{cu}}{V_{steel}+V_{cu}}$, $\rho_{steel}^{\prime }=\frac{\rho_{steel}}{\rho_{m}}$, $\rho_{cu}^{’}=\frac{\rho_{cu}}{\rho_{m}}$, δ_steel_ = (n − 1) * δ_t_, n represents the layer number and *δ_t_* is the layer thickness.

(13) $$R=\frac{\delta }{kA}$$

(14) $$R_{m}=\frac{\delta_{steel}}{k_{steel}A}+\frac{\delta_{cu}}{k_{cu}A}$$

(15) $$k_{m}=\frac{\delta_{Steel}+\delta_{Cu}}{\frac{\delta_{Steel}}{k_{Steel}}+\frac{\delta_{Cu}}{k_{Cu}}}$$

(16) $$\rho_{m}=\rho_{steel}V_{steel}^{\prime }+\rho_{cu}V_{cu}^{\prime }$$

(17) $$c_{m}=\rho_{steel}^{’}V_{steel}^{\prime }c_{steel}+\rho_{cu}^{\prime }V_{cu}^{\prime }c_{cu}$$

**Figure 6:**
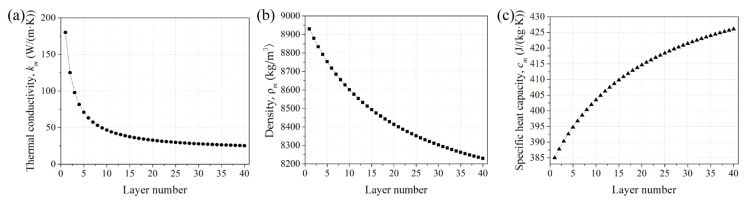
The calculated layer-dependent effective physical properties of the copper/steel bimetal: (**a**) effective thermal conductivity; (**b**) effective density; (**c**) effective specific heat capacity.

Table 1 provides the values of the single metal thermophysical properties of CuCr and the self-developed martensite stainless steel (07Cr15Ni5), respectively. Figure 6 shows the theoretically calculated layer-dependent effective thermophysical properties (effective thermal conductivity, density, and specific heat capacity) of the copper/steel bimetal according to Equations (14)–(16).

## 3. Printability Predictions

### 3.1. Printability Maps for the First Layer

During the DED process, the processing parameters such as laser power and scanning velocity determine the heat flow of laser energy to the powder and substrate and ultimately affect the temperature field. According to Equations (1) and (8), increasing laser power or decreasing scanning velocity transmits more energy to the powder and substrate. By increasing the heat input, fusion commences when the temperature of the material rises from the ambient temperature to the melting point (*T_m_*). Further increasing the laser energy will continue to raise the temperature to the boiling point (T_b_). According to reference [48], when the peak temperature (T_max_) of the molten pool reaches T_b_, the recoil pressure caused by the evaporation drives the molten pool to the keyhole mode. Therefore, the condition T_max_ = T_b_ is identified as the keyhole mode threshold.

**Figure 7:**
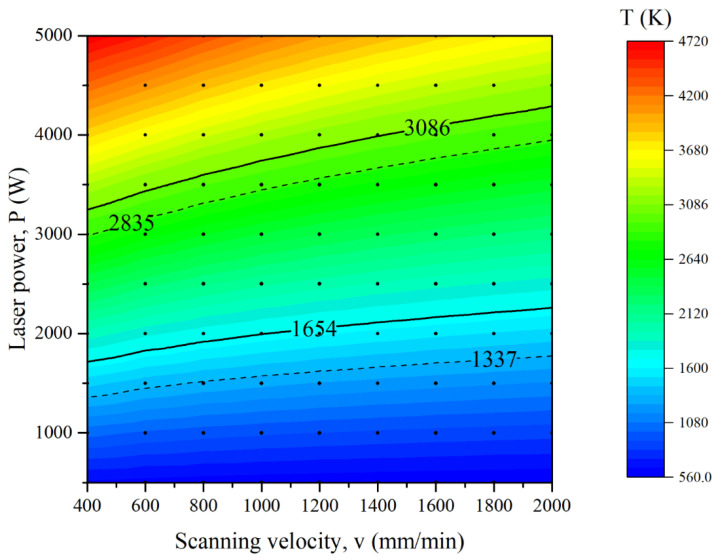
The peak temperature of different parameters for the first layer (T_s_cu_ = 1337 K, T_s_steel_ = 1654 K, T_b_cu_ = 2835 K, T_b_steel_ = 3086 K).

The printability map is determined by analyzing the temperature characteristics of the molten pool under various processing parameters. Table 2 shows the processing parameters calculated for the first layer. Figure 7 shows the peak temperature for different parameters combined with laser power and scanning velocity for the first layer. The isotherms of the melting and boiling points of copper and steel (T_s_cu_ = 1337 K, T_s_steel_ = 1654 K, T_b_cu_ = 2835 K, T_b_steel_ = 3086 K) are marked in Figure 7, respectively. According to the peak temperature at the center of the molten pool, three situations can be obtained: (1) T_max_ < T_s_steel_; (2) T_s_steel_ < T_max_ < T_b_cu_; (3) T_max_ > T_b_cu_. For situation (1), the temperature of the molten pool cannot reach the melting point of the steel (1654 K). Therefore, no fusion will occur because of the insufficient input energy. The steel coating cannot be fabricated on the copper substrate and form a metallurgical bond. For situation (2), the molten pool temperature is in the range of T_s_steel_ and T_b_cu_. Therefore, the fusion track can be obtained in the conduction mode. For situation (3), the molten pool temperature is higher than the T_b_cu_, and the fusion track can be obtained in the keyhole mode. Firstly, the parameters for situation (1) are excluded because no fusion occurred between the copper substrate and steel powder. Secondly, the keyhole mode can lead to a porosity void with vapor entrapping at the bottom of the molten pool [49,50] as well as a high dilution of the molten pool [51], which is not expected to produce good mechanical properties. Hence, the parameters for situation (3) are excluded. From the results of calculation and analysis above, situation (2) is evaluated as appropriate process maps before starting the experiments. Although the criterion of T_s_steel_ < T_max_ < T_b_cu_ may not be a precise condition, it can narrow the range of experimental parameters, and further parameters optimization can be conducted from the experiment based on the results. The peak temperature criterion has also been applied in the selective laser melting process [36,52].

### 3.2. Printability Maps for Multi-Layer

**Figure 8:**
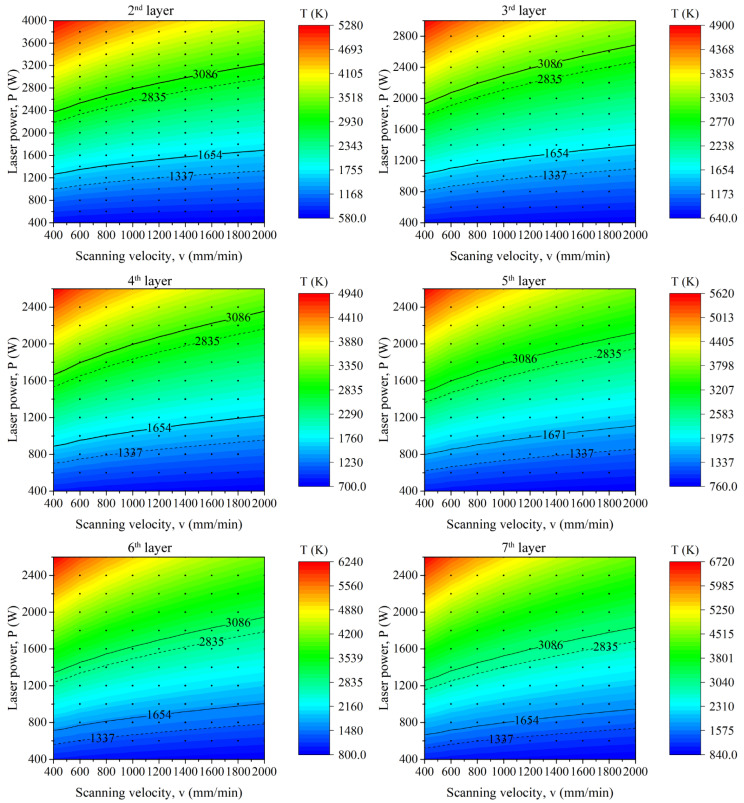
The peak temperature of different parameters for the 2nd to 7th layers.

**Figure 9:**
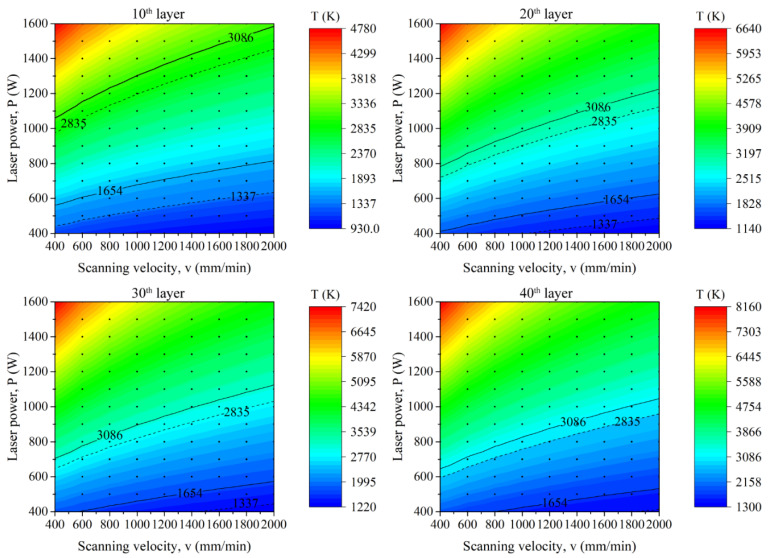
The peak temperature of different parameters for the 10th to 40th layers.

Since a layer of steel has been deposited on the copper substrate, the effective thermal conductivity, laser absorption, etc., will change significantly from the second layer. Therefore, the processing parameters will vary with the layer number. Figure 8 shows the calculated peak temperature distribution for the second to seventh layers. Similarly, the isotherms of the melting and boiling points of copper and steel are marked and different regions are labeled. As described in Figure 7, the peak temperature in the range of T_s_steel_ < T_max_ < T_b_cu_ is selected for the first layer. However, the deposited layer will be in direct contact with the steel from the second layer. Hence, the criteria for the upper limit of the peak temperature can change to the boiling point of the steel, i.e., T_max_ < T_b_steel_. Apparently, for a certain parameter combination with laser power and scanning velocity, the peak temperature increases with the layer number. Namely, the process maps move towards the low energy region and the printability maps become narrow. This phenomenon is more pronounced for the 10th to 40th layers, as is shown in Figure 9.

**Figure 10:**
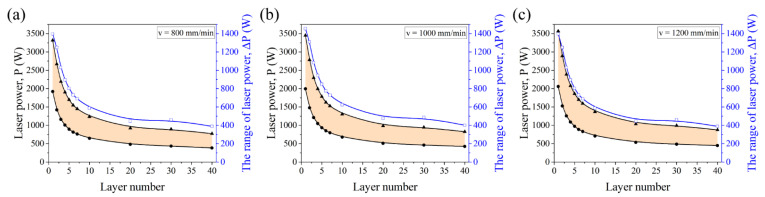
The variation and range of laser power with the layer number at different scanning velocities: (**a**) v = 800 mm/min; (**b**) v = 1000 mm/min, and (**c**) v = 1200 mm/min.

Figure 10 shows the optimal laser power maps dependent on the layer number when fixing the scanning velocity and hatching space. The range of laser power (ΔP) for a certain scanning velocity is defined as ΔP = P_max_ − P_min_ to evaluate the parameter range, where P_max_ and P_min_ represent the maximum and minimum laser power for the printability range. The scanning velocities ranging from 800 to 1200 mm/min are selected for the consideration of both the fabrication efficiency and the stability of powder feeding. It can be seen that the laser power changes in a large range as the layer number increases. Taking the scanning velocity of 800 mm/min as an example, the laser power should be in the range of 1920 to 3316 W for the first layer, and 384 to 773 W for the 40th layer. The decline rate of P_max_ and P_min_ is as high as 77 and 88% from the first layer to the 40th layer. Furthermore, the P_max_ and P_min_ decrease drastically for the first ten layers with the decline rate of 63 and 66% as the layer number increases and decreases slowly from the 10th to the 40th layer with the decline rate of 37 and 40%. In addition, Figure 10 also reveals a sharp fall of ΔP for the first ten layers and then a slight decline after ten layers, which corresponds to the variation of effective thermal conductivity with the number of layers from Figure 6a. The ΔP is 1395 W for the first layer and 389 W for the 40th layer with a decline rate of 72%. Compared to the high effective thermal conductivity for the first ten layers, the peak temperature response is more sensitive to the relatively low effective thermal conductivity because the energy can be conducted away quickly for the high effective thermal conductivity.

## 4. Verifications

### 4.1. Experiments

The initial material was self-developed martensite stainless steel (07Cr15Ni5). The argon gas atomized spherical steel powder has the average diameter of 28.9 µm. The CuCr alloy substrate with dimensions of 135 mm × 180 mm × 6 mm was rolled (900~950 °C) and annealed (400~450 °C) in the experiment. The chemical composition (wt.%) of the CuCr alloy substrate is 99.5 Cu and 0.5 Cr. The substrate surfaces were roughened, sandblasted, and cleaned with alcohol before the experiment. The DED system includes a 6 kW IPG YLR-6000 fiber laser (IPG laser GmbH, Burbach, Germany) with the beam size of 1 mm, a 6-axis robot, a powder feeder (HUST-III), and a self-made laser head. The 6-axis robot controls the movement of the laser head along the X–Y plane and/or the Z direction. The deposition area is protected from oxidation by the argon shielding gas. Details about the DED system have also been described previously [3,53]. The cross-section specimen was mechanically polished and then etched by a solution of 2 mL HF, 8 mL HNO_3_, and 90 mL H_2_O at room temperature to reveal the morphology of the multi-layer steel coatings.

### 4.2. Verification of the Single-Track Molten Pool Width

Due to the high energy input during the DED process, the temperature is difficult to measure directly. The molten pool widths are generally used for verification in thermal analysis, which is frequently adopted for the DED and SLM processes [54,55]. The molten pool width is obtained by the isothermal curves of the calculation according to the melting temperature of steel (1654 K) as depicted in Figure 3. Table 3 shows the processing parameters used for the single track.

**Figure 11:**
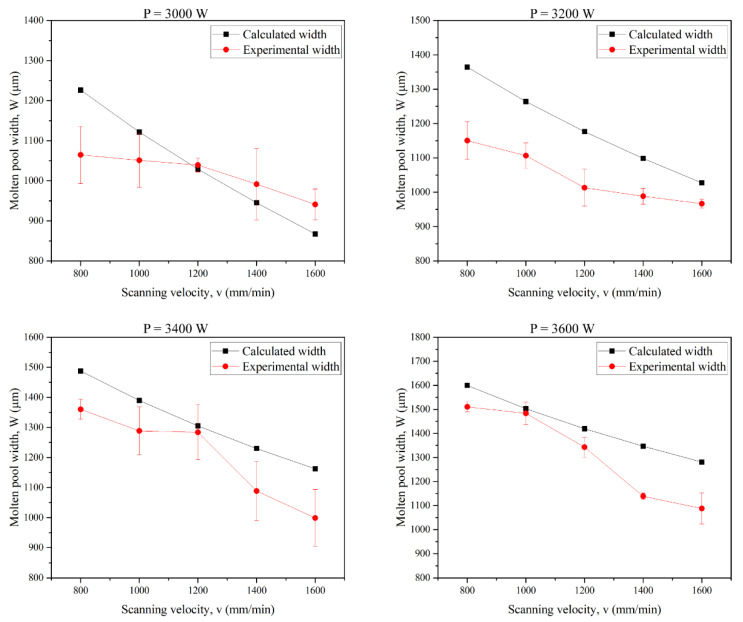
Comparison of experiment and calculated values of the single-track molten pool width.

As shown in Figure 11, the calculated molten pool width matches well with the experimental measurements. Both the calculated and experiment results show that the molten pool width decreases as the scanning velocity increases. According to Equations (9) and (10), the increasing scanning velocity reduces particle catchment efficiency, hence the energy delivered by powder to the substrate is reduced. On the other hand, high scanning velocity means a shorter contact duration of the laser with the substrate, therefore, less energy is absorbed directly by the substrate. As a result, the molten pool width decreases with the scanning velocity. Since heat loss by the molten pool flow and the latent heat of fusion are not taken into account, the calculated widths are slightly higher than the experimental measurements. However, the maximum relative error is 18.59%, which demonstrates the accuracy of the thermal model.

### 4.3. Verification of Printability Maps for the First Layer

**Figure 12:**
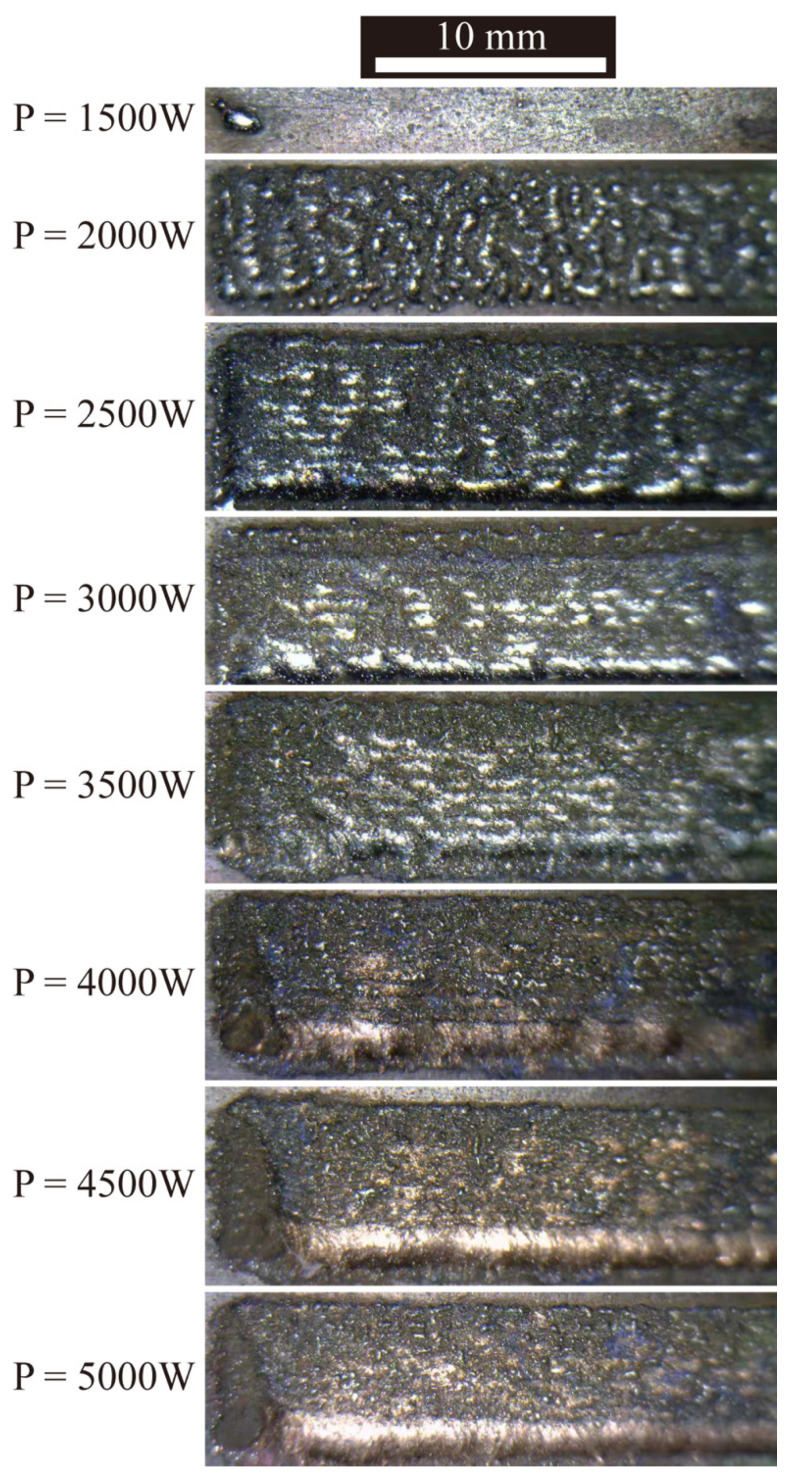
Surface morphologies of the single-layer specimens.

**Figure 13:**
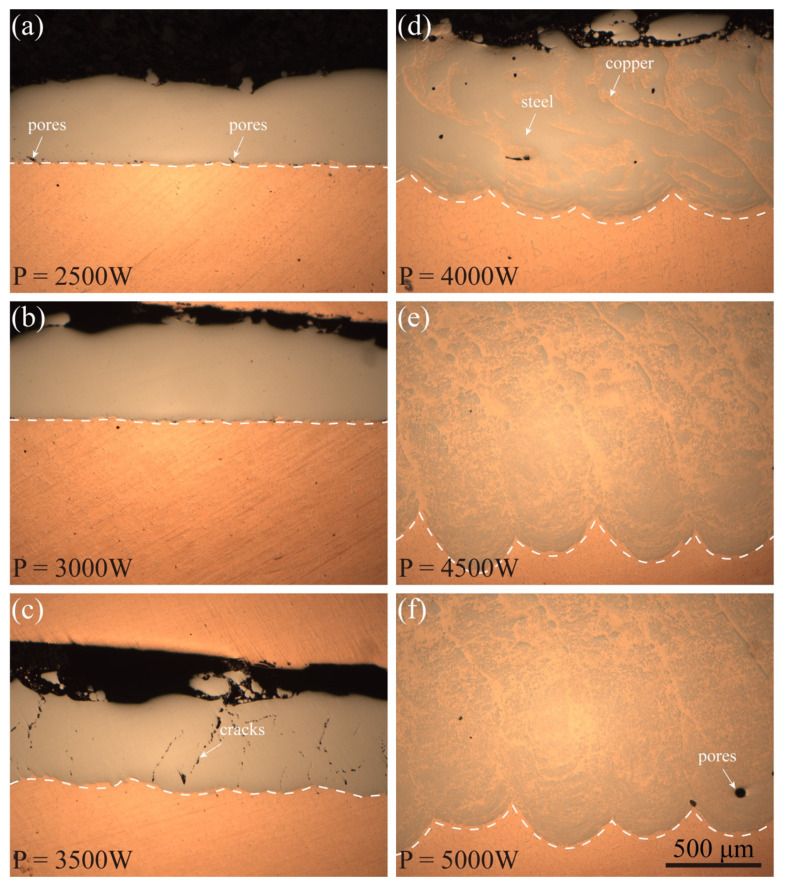
Cross-section morphologies of the single-layer specimens at different laser power: (**a**) P = 2500 W, (**b**) P = 3000 W, (**c**) P = 3500 W, (**d**) P = 4000 W, (**e**) P = 4500 W, and (**f**) P = 5000 W.

To verify the analytical model for the first layer, the laser scanning velocity was fixed at 800 mm/min and the scanning patch at 0.3 mm. As illustrated in Figure 12, the influence of laser power on the solidification behavior of the fusion track was investigated. Because of the inadequate energy, no scanning track emerged on the substrate when the laser power was 1500 W. Fusion layers can be observed when the laser power is higher than 2000 W. As shown from the cross-section of the first layer in Figure 13, the fusion tracks are formed in the conduction mode with laser power of 2500 W~3500 W, and the morphology is uniform. When increasing the laser power to 4000 W~5000 W, the fusion tracks are formed in the keyhole mode with heterogeneous morphology. According to the calculated results above, fusion tracks can be formed in the conduction mode with the laser power of 1920 W~3316 W at the fixed scanning speed of 800 mm/min. The molten pool should be the keyhole mode with the laser power of above 3316 W. However, from the cross-section morphology in Figure 13, conduction mode is observed when the laser power is 3500 W. Because heat loss by the molten pool flow and the latent heat of fusion is not taken into account, the peak temperatures may be overpredicted slightly.

According to the experimental results shown in Figure 13, although the molten pool can be formed at 2000 to 3500 W, the forming quality is still different. When P = 2000 W, the laser energy is too low and the fused layer cannot be formed continuously. When the laser power P = 2500 W, holes can be observed near the interface with fusion in place. When the laser power is increased to P = 3000 W, the molten layer can be well formed and no cracks are produced with the flat and straight copper–steel interface, and no obvious semi-elliptical molten pool is seen, which means the melting area is small. When the laser power increases to P = 3500 W, a semi-elliptical molten pool can be observed at the interface and cracks can be observed. It should be noted that this model can only predict formability and narrow the range of experimental parameters before conducting experiments, while defects such as cracks cannot be predicted and further experimental studies are needed.

### 4.4. Verification of Printability Maps for Multi-Layer

**Figure 14:**
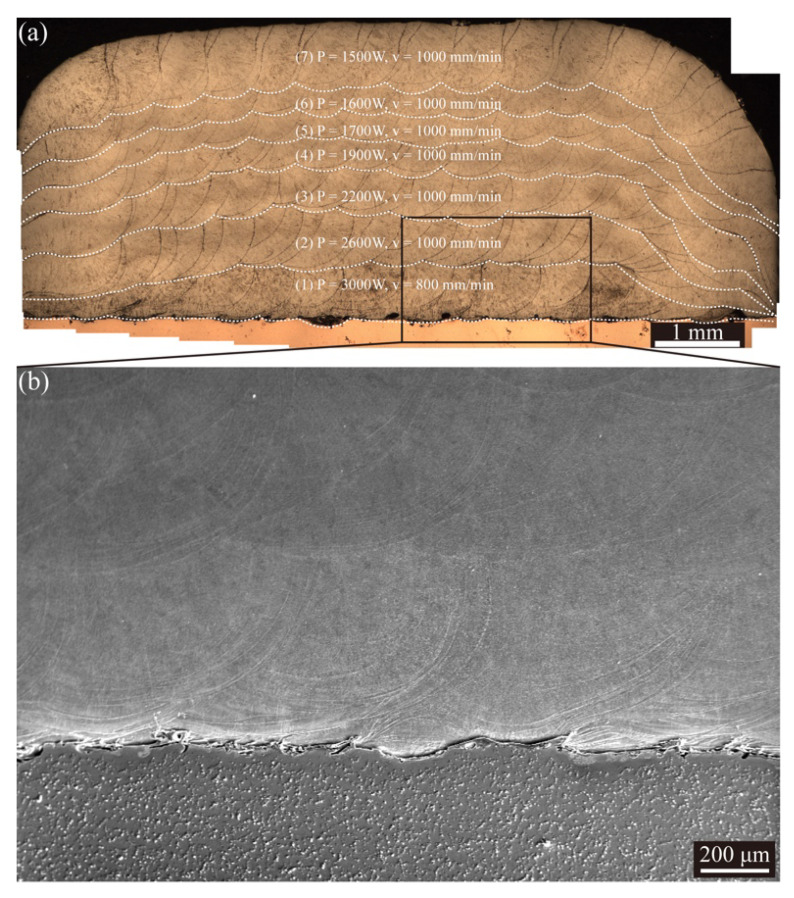
Cross-section morphologies of the seven-layer specimen: (**a**) overview of the cross-section; (**b**) magnified view of the copper/steel interface.

**Figure 15:**
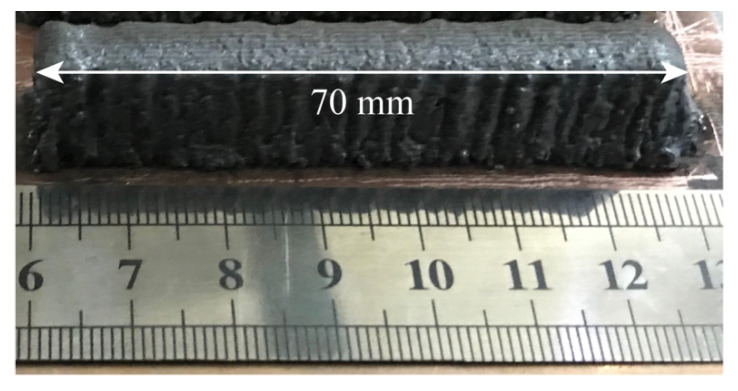
Morphology of the bulk specimen.

The seven-layer specimen was fabricated based on the calculated results. The parameters used for the seven-layer specimen and bulk specimen are listed in Table 4. Figure 14a shows the cross-section morphologies of the seven-layer specimen. It should be noted that because the copper/steel interface with the mixed Cu-rich phase and Fe-rich phase is more sensitive to the etching agent, the aggressively etched areas at the interface will look like pores. In fact, the fabricated specimen is free of obvious defects, which can be confirmed compared with the unetched sample without obvious defects at the interface from Figure 13b. The interface of the copper/steel shown in Figure 14b indicates that a good copper/steel interface without cracks was obtained. Furthermore, the 40-layer bulk specimen with dimensions of about 70 × 8 × 12 mm^3^ was fabricated as shown in Figure 15.

## 5. Conclusions

A layer-dependent analytical model is established to predict the layer-dependent processing parameters when fabricating the 07Cr15Ni5 steel on the CuCr substrate at the fixed layer thickness (0.3mm) and hatching space (0.3mm) and agrees well with experiments. Based on the research, the main conclusions can be drawn as follows:

(1) Considering both the great difference in thermophysical properties between copper and steel and the layer-based fabrication character of the AM process, the evolution of the effective thermophysical properties, including the effective thermal conductivity, effective density, and effective specific heat capacity with layer number are calculated. Meanwhile, the changes in absorption rate and catchment efficiency with the processing parameters are also taken into account. Then the layer-dependent effective material properties and processing parameters are provided to the Cline–Anthony thermal model. Thus, the layer-dependent analytical model is established.
(2) The model is applied to predict the printability maps as the deposition layer increases. The laser power decreases drastically for the first ten layers as the layer number increases and decreases slowly from the 10th to the 40th layer when other processing parameters are fixed. The decline rate of the maximum and minimum laser power for the printability range is as high as 77 and 88% from the first layer to the 40th layer at the scanning velocity of 800 mm/min. In addition, the ΔP is 1395 W for the first layer and 389 W for the 40th layer with a decline rate of 72%. The significant narrowing of the printability maps with the layer number is due to the decreases in effective thermal conductivity and effective specific heat capacity as well as the increase in effective density.
(3) The calculated results based on the proposed analytical model agree well with the experiments. The maximum relative error for the single-track molten pool width between the calculated and experimental results is 18.59%. Furthermore, the calculated formability of the first layer and multi-layer is in agreement with the experiment. The defects-free bulk specimen with dimensions of about 70 × 8 × 12 mm^3^ is successfully fabricated according to the predicted printability maps.

The proposed analytical model provides a new guidance to determine the processing windows for novel multi-material components, especially for the multi-materials whose physical properties are significantly different. Nevertheless, the criterion for the good fusion tracks is based on the peak temperature of the molten pool in the range of T_s_steel_ ~ T_b_steel_, which may not be a precise condition to determine the final process maps. More rigorous criteria related to molten pool defects such as pores and balling formation are to be identified to further narrow the process map. Furthermore, HS and m_p_ also have a significant impact on printability during the fabrication of multi-material components, which will be discussed in the future.

## Figures and Tables

**Table 1:** Thermophysical properties of CuCr and steel for the calculation [43,44,45,46,47].

Parameters	CuCr	Steel
Density, ρ (kg m^−3^)	8.90	7.78
Melting point, *T_m_* (K)	1358	1654
Boiling point, *T_b_* (K)	2835	3086
Thermal conductivity, *k* (W m^−1^ K^−1^)	180	80
Specific heat capacity, *c* (J kg^−1^ K^−2^)	385	450
Laser beam absorptivity, β	0.2	0.5

**Table 2:** Processing parameters for the first layer.

Parameters	Values
Laser power (P, W)	500~5000 (500 increment)
Scanning velocity (V, mm/min)	400~2000 (200 increment)
Hatching space (HS, mm)	0.3
Powder feeding rate (g/min)	12
Z-axis increment (mm)	0.3
Laser spot diameter (mm)	1

**Table 3:** Processing parameters for the single track.

Parameters	Values
Laser power (P, W)	3000~3600 (200 increment)
Scanning velocity (V, mm/min)	800~1600 (200 increment)
Powder feeding rate (g/min)	12
Laser spot diameter (mm)	1

**Table 4:** Processing parameters used for the seven-layer and bulk specimen.

Layer Number	Laser Power (P, W)	Scanning Velocity (V, mm/min)
1	3000	800
2	2600	1000
3	2200	1000
4	1900	1000
5	1700	1000
6	1600	1000
7	1500	1000
10	1300	1000
20	950	1000
30	900	1000
40	800	1000
